# Psychological impact and implementation of preventative measures in hemodialysis centers during the COVID-19 pandemic: a provincial questionnaire survey in China

**DOI:** 10.1007/s11255-021-02875-x

**Published:** 2021-06-30

**Authors:** Yuanhan Chen, Yanhua Wu, Penghua Hu, Xia Fu, Shuangxin Liu, Li Song, Wei Dong, Xueqing Yu, Xinling Liang

**Affiliations:** 1 Division of Nephrology, Guangdong Provincial People’s Hospital, Guangdong Academy of Medical Sciences, No.106 Zhongshan Road 2, Guangzhou, 510080 Guangdong China; 2 Guangdong Provincial Geriatrics Institute, Guangzhou, 510080 China

**Keywords:** COVID-19, Psychological status, Hemodialysis centers, Preventative measures, Questionnaire survey

## Abstract

**Objectives:**

This study investigated the psychological status of patients and staff, and the implementation of preventative measures in hemodialysis centers in Guangdong province, China, during the 2019 novel coronavirus disease (COVID-19) pandemic.

**Methods:**

An electronic questionnaire survey was carried out anonymously between March 28 and April 3, 2020. All of the 516 hemodialysis centers registered in Guangdong province were invited to participate in the survey. The questionnaires were designed to investigate the psychological status of hemodialysis patients and general staff members (doctors, nurses, technicians, and other staff), and to address the implementation of preventative measures for administrators (directors or head nurses) of the hemodialysis centers.

**Results:**

A total of 1782 patients, 3400 staff, and 420 administrators voluntarily participated in this survey. Patients living in rural areas reported a higher incidence of severe anxiety compared to those living in other areas (in rural areas, towns, and cities, the incidence rate was 17.0%, 9.0%, and 8.9%, respectively, *P* < 0.001). Medical staff were less likely to worry about being infected than non-medical staff (13.1% vs 30.3%, respectively, *P* < 0.001). With respect to the implementation of preventative measures, hemodialysis centers in general hospitals outperformed stand-alone blood purification centers, while tertiary hospitals outperformed hospitals of other levels. However, restrictions regarding the admission of non-resident patients were lower in tertiary hospitals than in other hospitals. In this situation, only one patient imported from Hubei province was diagnosed with COVID-19.

**Conclusions:**

COVID-19 did not significantly affect the psychological status of most patients and medical staff members. Due to the implementation of comprehensive preventative measures, there were no cluster outbreaks of COVID-19 in hemodialysis centers. This provincial-level survey may provide referential guidance for other countries and regions that are experiencing a similar pandemic.

## Introduction

From December 2019, the first case of the 2019 novel coronavirus disease (COVID-19) was reported in Wuhan in Hubei province, China. This disease rapidly spread worldwide and caused a devastating pandemic [1–4]. Hemodialysis patients belong to a special population and they are at a very high risk of infection [5, 6].

Guangdong province in southern China has a large population of over 100 million people, and imbalances in economic and medical development are still evident across provinces. Across Guangdong province, there are nearly 50,000 online hemodialysis patients at 516 hemodialysis centers. The most developed regions in China, such as the Pearl River Delta region, and poor or less-developed regions coexist. Given this diversity, an examination of the psychosocial epidemiology and preventative experience of Guangdong should provide valuable information for the battle against COVID-19.

By March 31, 2020, a total of 81,554 confirmed cases of COVID-19 (including 67,802 in Hubei province) and 3312 deaths (including 3193 in Hubei province) had been reported in China [7], and 1501 cases and eight deaths had been reported in Guangdong [8]. Although the reporting rates of COVID-19 in Guangdong were significantly lower than in Hubei, the COVID-19 risk to hemodialysis patients in Guangdong was similar to that observed in cities close to Wuhan, the epicenter of COVID-19 in China. A recent survey showed that the prevalence of serological positive of COVID-19 antibodies in hemodialysis patients from the two provinces was similar, 2.8% in Guangdong and 3.6% in Hubei. In addition, the floating population of Guangdong was significant, especially during the Spring Festival travel rush, which overlapped the COVID-19 outbreak. Thus, effective control of COVID-19 among the hemodialysis patient population remains a huge challenge. This stress would inevitably cause medical staff and patients to suffer from anxiety.

To prevent COVID-19 in hemodialysis centers, the Chinese Society of Nephrology published recommendations for the prevention and control of the novel coronavirus disease in blood purification centers in February 2020[9], which was based on a series of guidelines for the diagnosis and treatment of COVID-19 published by the National Health Commission of China [10]. These recommendations provide a standard principle for managing hemodialysis patients during the pandemic. However, only limited amounts of data are available regarding the feasibility and implementation of these recommendations.

We conducted a provincial questionnaire-based survey to investigate the psychological status and implementation of preventative measures in response to COVID-19 outbreaks in all of the registered hemodialysis centers.

## Methods

An anonymous electronic questionnaire survey was distributed using an Internet survey platform (https://www.wjx.cn/) between March 28 and April 3, 2020. Three questionnaires were designed for hemodialysis patients, general staff (doctors, nurses, technicians, and other staff) and administrators (directors or head nurses; no more than two persons in each center), respectively. All of the administrators from the 516 registered hemodialysis centers in Guangdong province were invited to respond to the questionnaire survey voluntarily through WeChat, the most popular web-based social media application in China. The administrators who had responded then invited staff and patients to participate in the respective questionnaire. All of the respondents were voluntarily requested to complete the questionnaires, and it was explained that the results of the survey should not be used as a reference for medical quality evaluation purposes.

### Questionnaires design

In respect to the design of the questionnaires, the primary questionnaire was modified by a dialysis quality control specialist. The questionnaire included demographic information, education, occupation, income and psychological states, which were evaluated using a semi-quantitative method. Primary preventative measures included staff protection, management of patients and patient escorts, preview triage, disinfection and isolation, and strengthening the management of medical waste materials. The implementation of preventative measures was self-evaluated by administrators.

Two questionnaires were designed to investigate the psychological status of hemodialysis patients and staff (doctors, nurses, technicians, and other staff), respectively. An additional questionnaire was designed for the administrators (directors or head nurses) of hemodialysis centers, so as to address the implementation of preventative measures, including strengthened patient triage management, restricting caregiver visits to patients during dialysis, strengthened prevention amongst staff, and improved patient education and protection. To standardize the answers, multiple choice questionnaires were designed. For data protection purposes, citizen identification numbers or addresses were not included.

### Statistical analysis

Statistical analysis was performed using SPSS version 25.0 software. Age, as a non-normally distributed variable, is expressed as the median (25th percentile, 75th percentile), and the comparison between groups was tested using the rank-sum test. Count data are expressed as the number of cases (%), and the difference between groups was compared using the chi-squared test or Fisher’s exact test A two-tailed *P* value < 0.05 was set as statistically significant.

Data were expressed as a percentage of positive answers for each question. To better describe the differences between the levels of hospitals, respondents were subdivided on the basis of dialysis centers in general hospitals and stand-alone dialysis centers. General hospitals were further classified as primary, secondary, or tertiary.

## Results

### Questionnaire responses

A total of 1822 hemodialysis patients, 3455 general staff, and 600 administrators took part in the survey. Respondents’ questionnaires were excluded due to repetition or incompleteness in responses. Finally, 1782 patient questionnaires, 3400 staff questionnaires, and 420 administrator questionnaires were analyzed.

### Patient responses

Of the 1782 patients who participated in the questionnaire, 500 (28.1%) were from rural areas, 266 (14.9%) from townships, and 1016 (57.0%) from cities (Table 1).

**Table 1:** Responses from the patients

	Number (*n* = 1782)	Percentage %
Gender		
Male	1022	57.4
Female	760	42.6
Location		
Urban	1016	57.0
Township	266	14.9
Rural	500	28.1
Monthly income		
Less than 3000 RMB	1154	64.8
3000–5000 RMB	382	21.4
5001–10,000 RMB	179	10.0
Above 10,000 RMB	67	3.8
Type of medical care		
Free medical care	51	2.9
Urban employee basic medical insurance	830	46.6
Urban resident basic medical insurance	516	29.0
New rural cooperative medical scheme	295	16.6
Others	90	5.1
Caregiver		
Parents	144	8.1
Spouse	989	55.5
Offspring	314	17.6
Brother or sister	30	1.7
Friend	10	0.6
Nursing worker	17	1.0
Alone	278	15.6
Impact of pandemic prevention and control on your life		
No influence	264	14.8
Slight influence	999	56.1
Moderate influence	295	16.6
Great influence	224	12.6
Worrying about being infected with COVID-19 when going to hospital for dialysis		
Great worry	398	22.3
Moderate worry	348	19.5
Slight worry	730	41.0
Not worry at all	306	17.2
Feeling anxious and the extent of anxiety during the COVID-19 pandemic		
Severe anxiety	199	11.2
Moderate anxiety	716	40.2
No or occasional anxiety	867	48.7
Satisfaction with the protective measures implemented		
Satisfied	1572	88.2
Neither satisfied nor dissatisfied	199	11.2
Not satisfied	11	0.6

A total of 199 patients (11.2%) reported serious anxiety. Among them, 85 (42.7%) patients were from rural areas, 24 (12.1%) were from townships, and 90 (45.2%) were from urban areas. The rate of serious anxiety was 17.0%, 9.0%, and 8.9%, respectively (*P* < 0.001) (Table 2), indicating serious levels of anxiety in rural areas. This tendency was further confirmed by the analysis stratified by occupation that farmers had highest rate of anxiety (*P* < 0.001). In addition, lower education levels and higher medical financial stress were associated with anxiety (*P* < 0.001) (Table 2). Moreover, 1476 (82.8%) patients reported that they were concerned about the risk of COVID-19 infection when attending the hospital to receive dialysis treatment, and 398 (22.3%) of these patients reported that they were very anxious. However, 1572 patients (88.2%) reported that they were satisfied with the preventative measures.

**Table 2:** The relationship between clinical characteristics and psychology

	Non-anxiety	Anxiety	X2/M score	*p* values
Gender			0.869	0.351
Male,* N* (%)	914(89.4)	108(10.6)		
Female,* N* (%)	669(88.0)	91(12.0)		
Age, median(Q25, Q75)	50(41–64)	50(40–62)	-0.656	0.512
Education			8.074	0.018
Junior high school or below,* N* (%)	816(87.0)	122(13.0)		
Senior high school,* N* (%)	431(89.8)	49(10.2)		
University degree or above,* N* (%)	336(92.3)	28(7.7)		
Marriage			0.137	0.987
Divorced,* N* (%)	79(89.8)	9(10.2)		
Widowed,* N* (%)	81(88.0)	11(12.0)		
Unmarried,* N* (%)	166(88.8)	21(11.2)		
Married,* N* (%)	1257(88.8)	158(11.2)		
Location			23.841	< 0.001
Rural,* N* (%)	415(83.0)	85(17.0)		
Township,* N* (%)	242(91.0)	24(9.0)		
Urban,* N* (%)	926(91.1)	90(8.9)		
Social functions			35.022	< 0.001
Employee,* N* (%)	262(94.2)	16(5.8)		
Farmers,* N* (%)	411(82.2)	89(17.8)		
Retired workers,* N* (%)	449(91.8)	40(8.2)		
Others,* N* (%)	461(89.5)	54(10.5)		
Monthly income			7.331	0.062
Less than 3000 RMB,* N* (%)	1009(87.4)	145(12.6)		
3000–5000 RMB,* N* (%)	350(91.6)	32(8.4)		
5001–10,000 RMB, *N* (%)	165(92.2)	14(7.8)		
Above 10,000 RMB,* N* (%)	59(88.1)	8(11.9)		
Type of medical care			16.828	0.001
Free medical care, *N* (%)	48(94.1)	3(5.9)		
Basic medical insurance,* N* (%)	1213(90.1)	133(9.9)		
New rural cooperative medical scheme,* N* (%)	251(85.1)	44(14.9)		
Others, *N* (%)	71(78.9)	19(21.1)		

### Staff responses

Among the 3400 staff members who participated in the questionnaire, 588 (17.3%) were doctors, 2703 (79.5%) were nurses, and the remaining staff members 109 (3.2%) comprised non-medical workers (Table 3).

**Table 3:** Responses from the staffs

	Number (*n* = 1782)	Percentage %
Occupation		
Doctor	588	17.3
Nurse	2703	79.5
Non-health care worker	109	3.2
Working shifts in a fever clinic, emergency department or infectious diseases department (bedside CRRT)	308	9.1
Workload changed compared with before		
Reduced relatively	163	4.8
Unchanged	633	18.6
Increased slightly	1507	44.3
Increased substantially	1097	32.3
Symptoms associated with COVID-19 infection		
Respiratory rate increased, or dyspnea	26	0.8
Cough	14	0.4
Fever for more than 72 h	4	0.1
Fatigue and anorexia	80	2.4
No above symptoms	3303	97.1
Insufficient self-protection when contacting patients with confirmed or suspected COVID-19 (such as without wearing masks)	53	1.6
Worrying about getting infected		
Not at all	530	15.6
Somewhat worry	2406	70.8
Very worry	464	13.6
Affection of intensity of the workload during the pandemic		
The influence is so great that it is difficult to persist to work	45	1.3
Great effect, but can persist	1207	35.5
Marginal effect	1390	40.9
No effect	758	22.3

COVID-19 significantly increased staff workloads. More than three-quarters of the staff (2604, 76.6%) reported an increased workload and 1097 (32.3%) reported a much higher workload. Workloads were associated with anxiety. The reported rates of worry about the risk of COVID-19 infection were 20.1%, 11.0%, 10.1%, and 8.6% among staff who reported that their workload had substantially increased (*n* = 220), slightly increased (*n* = 166), was unchanged (*n* = 64), or had somewhat decreased (*n* = 14), respectively. A total of 464 staff members were seriously concerned about the risk of infection, including 33 (30.3%) non-medical workers and 431 (13.1%) medical staff members (*P* < 0.001). It was noticeable that the staff members who were transferred from the fever clinic, emergency department, or infectious disease department were less worried about the risk of infection (*n* = 21, 6.8%) compared to those who worked in other departments (*n* = 443, 14.3%). In addition, medical knowledge seemed to be associated with a lower psychological burden. The reported rates of difficulty associated with having to continue to work due to heavy workloads were 0.9% among doctors (n = 5), 1.2% among nurses (n = 33), and 6.4% among non-medical workers (*n* = 7). Moreover, 53 staff members (1.6%) reported that they had contact with patients with confirmed or suspected COVID-19 infection, and this contact occurred without the implementation of adequate prevention and protective measures.

### Administrator responses

Among the 420 administrators who participated in the questionnaire, 189 (45.0%) were from tertiary hospitals, 174 (41.4%) were from secondary hospitals, and 35 (8.3%) were from primary hospitals; 398 were from hospital hemodialysis centers and 22 (5.2%) were from stand-alone hemodialysis centers (Table 4).

**Table 4:** Responses from the administrators

	Number (*n* = 1782)	Percentage %
Level of hospital		
Tertiary hospital	189	45.0
Secondary hospital	174	41.4
Primary hospital	35	8.3
Independent dialysis center	22	5.2
Location of hospital		
Guangzhou or Shenzhen city	130	31.0
Other regions in Guangdong Province	290	69.0
Any staff unable to work due to excessive panic in your department	5	1.2
Self-evaluation the ability to control the infection in your administrator		
Adequate prevention and control measures taken at hemodialysis center	384	91.4
Coordination of hospital management	362	86.2
Staff with strong awareness of disease prevention and control	379	90.2
Implement the following prevention and control strategies		
Strengthened patient triage management	413	98.3
Restricting caregiver visits to patients during dialysis	406	96.7
Strengthened prevention and disease control amongst staff	407	96.9
Improved patient education and protection	413	98.3
Restricting acceptance of non-resident patients	396	94.3
The level of prevention and control for this outbreak		
Prevention and control as usual	12	2.9
Prevention and control as required by higher authorities	122	29.0
Actively strengthen prevention and control	286	68.1
Any staff members or patients infected COVID-19	2	0.5

**Fig. 1:**
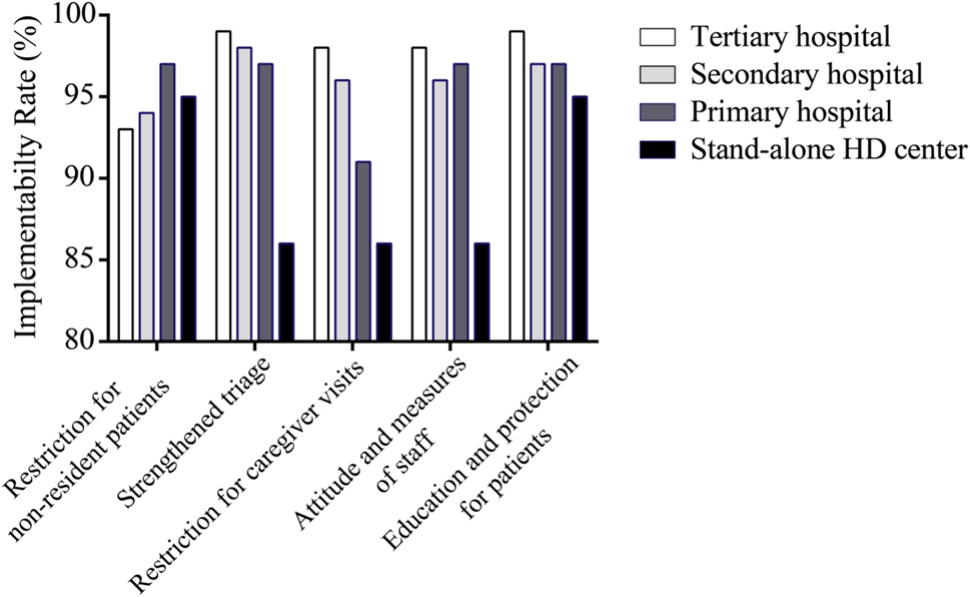
Implementation of preventative measures

The reported rates of adequate preventative measures, correct attitudes towards prevention, and integration across management departments were 91.4%, 90.2%, and 86.2%, respectively. In particular, two pregnant staff members were unable to work due to excessive panic. These self-evaluation results represent a good and comprehensive implementation of preventative measures. We then analyzed the preventative measures which were stratified based on the level of the hemodialysis centers. The rates of restriction of admitted non-resident patients were high among all of the centers at different levels (Fig. 1). The implementation of preventive measures showed a decreasing trend from hemodialysis centers at tertiary hospitals, to secondary hospitals and primary hospitals, to stand-alone hemodialysis centers, with respect to strengthened triage, restricting caregiver visits during dialysis treatment, an attitude of respecting preventative measures among staff, and improvements in the education and protection of patients (Fig. 1).

### Infection of COVID-19 in hemodialysis centers

In all of the hemodialysis centers in Guangdong province, one case of COVID-19 was confirmed. This case was imported from Hubei province and it was first diagnosed in Guangdong province. The patient was immediately transferred to a designated hospital and no other patients or staff members were infected.

## Discussion

In this provincial questionnaire-based survey of 516 hemodialysis centers, the COVID-19 pandemic did not significantly affect the psychological status of hemodialysis patients and staff members. Preventative measures were implemented satisfactorily at all centers at each level, particularly centers in tertiary hospitals.

Our results indicated that patients from rural areas had a higher proportion of severe anxiety compared to those from other areas. This disparity may be associated with the unbalanced distribution of medical resources between urban and rural areas. Many rural areas lack dialysis centers. As such, some patients must travel a long distance to receive dialysis treatment. Travel was difficult during the COVID-19 outbreak due to the national travel restriction policy. In addition, most rural patients have lower incomes, which might exacerbate their anxiety. Thus, efforts should be made to improve the availability of psychological and travel assistance, particularly for patients living in rural areas.

Despite the fact that most staff members reported an increase in their workloads, this did not significantly exacerbate the psychological stress of staff, especially medical staff. Although fever clinics, emergency departments, and infectious disease departments are at a high risk of infection, less staff members who had transferred from these departments worried about the risk of infection, compared to staff members who did not work in these departments. According to the preventive policy of China, staff members who work in high-risk units are selected volunteers. The government provided various supports to these staff members in spiritual and material ways. These measures can help to relieve psychological stress. In particular, pregnant staff members are a population that is vulnerable to psychological stress. Humanistic care, emotional counseling and work transfers, when necessary, will be important for these specific populations.

The results revealed that medical staff had a better psychological health status than non-medical staff members, which was similar to the findings of previous studies that investigated the SARS outbreak [11]. However, the results conflicted with those of another nationwide study in China that revealed that medical health workers had a higher prevalence of mental health and psychosocial problems than nonmedical staff in hospitals during the COVID-19 outbreak [12]. A possible reason for this finding is that medical staff members are trained and have more medical knowledge about how to prevent infection. Another explanation might be attributed to their work intensity or environment [13, 14]. The other explanation for the disparity might be attributed to the geographic differences associated with these studies. Our survey was performed in Guangdong, where the SARS outbreak in 2003 offered insight into preventative experience. These findings highlight the importance of training, improving the work environment, and enhancing the stress management resources that are made available to non-medical staff members.

The centers at all levels implemented similar measures with respect to the restriction of non-resident patients, which indicated that the restriction for pandemic prevention was implemented adequately in Guangdong. Tertiary hospitals have better medical resources. On the other hand, they are located in cities, where a high level of pressure regarding population mobility exists. These features determine the leading role of tertiary hospitals in pandemic prevention. There are also privately operated stand-alone hemodialysis centers in Guangdong. Our results revealed that stand-alone centers had less prevention resources and the preventive measures were relatively inadequate. Thus, the superior quality control department should strengthen the guidance, assistance and supervision of these centers.

Because the questions were related to the medical quality of individual hemodialysis centers, this study was designed to safeguard anonymity to improve the reliability of responses. However, the results were still based on a subjective self-report method and parts of the questions were not standardized. Thus, it may not have been possible to avoid intra-individual variations and exaggeration regarding preventative measures. The other limitation concerns the non-randomized distribution of the questionnaire, which could result in selection bias. However, 600 administrators participated in the survey. Because the administrator number in a center was not more than two, the number of administrators who responded was more than half of the 516 registered hemodialysis centers in whole province. Therefore, the results of this survey are representative features of Guangdong Province.

## Conclusions

The overall psychological status of hemodialysis patients and staff members was stable during the COVID-19 pandemic in Guangdong. Tertiary hospitals played a leading role in prevention. These findings may provide practical guidance for other countries and regions that encounter similar pandemic experiences.
